# Multimodal Gait Abnormality Recognition Using a Convolutional Neural Network–Bidirectional Long Short-Term Memory (CNN-BiLSTM) Network Based on Multi-Sensor Data Fusion

**DOI:** 10.3390/s23229101

**Published:** 2023-11-10

**Authors:** Jing Li, Weisheng Liang, Xiyan Yin, Jun Li, Weizheng Guan

**Affiliations:** 1 School of Mechanical Engineering and Hubei Modern Manufacturing Quality Engineering Key Laboratory, Hubei University of Technology, Wuhan 430068, China; 2 School of Mechanical and Electrical Engineering, University of Electronic Science and Technology of China, Chengdu 611731, China; 3 Detroit Green Technology Institute, Hubei University of Technology, Wuhan 430068, China; 2011611212@hbut.edu.cn (J.L.); 2011611222@hbut.edu.cn (W.G.)

**Keywords:** neurodegenerative diseases, gait abnormality recognition, multi-sensor, time–frequency plots, CNN-BiLSTM network

## Abstract

Global aging leads to a surge in neurological diseases. Quantitative gait analysis for the early detection of neurological diseases can effectively reduce the impact of the diseases. Recently, extensive research has focused on gait-abnormality-recognition algorithms using a single type of portable sensor. However, these studies are limited by the sensor’s type and the task specificity, constraining the widespread application of quantitative gait recognition. In this study, we propose a multimodal gait-abnormality-recognition framework based on a Convolutional Neural Network-Bidirectional Long Short-Term Memory (CNN-BiLSTM) network. The as-established framework effectively addresses the challenges arising from smooth data interference and lengthy time series by employing an adaptive sliding window technique. Then, we convert the time series into time–frequency plots to capture the characteristic variations in different abnormality gaits and achieve a unified representation of the multiple data types. This makes our signal processing method adaptable to several types of sensors. Additionally, we use a pre-trained Deep Convolutional Neural Network (DCNN) for feature extraction, and the consequently established CNN-BiLSTM network can achieve high-accuracy recognition by fusing and classifying the multi-sensor input data. To validate the proposed method, we conducted diversified experiments to recognize the gait abnormalities caused by different neuropathic diseases, such as amyotrophic lateral sclerosis (ALS), Parkinson’s disease (PD), and Huntington’s disease (HD). In the PDgait dataset, the framework achieved an accuracy of 98.89% in the classification of Parkinson’s disease severity, surpassing DCLSTM’s 96.71%. Moreover, the recognition accuracy of ALS, PD, and HD on the PDgait dataset was 100%, 96.97%, and 95.43% respectively, surpassing the majority of previously reported methods. These experimental results strongly demonstrate the potential of the proposed multimodal framework for gait abnormality identification. Due to the advantages of the framework, such as its suitability for different types of sensors and fewer training parameters, it is more suitable for gait monitoring in daily life and the customization of medical rehabilitation schedules, which will help more patients alleviate the harm caused by their diseases.

## 1. Introduction

The global phenomenon of rapid population aging is undeniable. According to the World Health Organization (WHO), the number of individuals aged 60 and over surged from 1 billion in 2020 to 1.4 billion in 2022, with a projected increase to 2.1 billion by 2050. This demographic shift is profoundly impacting various domains, particularly in the realm of health [1]. The surging aging population is accompanied by a notable rise in individuals grappling with neurological diseases. These conditions often result in disability and cognitive decline, posing a significant threat to individuals’ physical well-being and amplifying the societal burden. Statistics reveal that neurological diseases contribute to 13.3% of total disability-adjusted life years (DALYs) and account for 19.5% of total deaths in the European region [2].

Neurodegenerative diseases often manifest with cognitive decline, gait issues, and balance deficits. Gait abnormalities arising from neurological diseases exhibit distinct characteristics in terms of gait dynamics [3]. For instance, Parkinson’s disease may manifest as a slowing of gait speed and rhythm [4], while amyotrophic lateral sclerosis (ALS) shows stride instability and reduced walking speed. Recognizing these unique gait patterns allows for the differentiation of various neurological diseases. Thus, leveraging gait-abnormality-recognition technology enables the early detection and distinction of signs associated with these diseases, providing an opportunity for timely interventions to mitigate the progression of neurological conditions.

For individuals dealing with neurological diseases, the technology of gait abnormality recognition offers healthcare providers additional insights into symptoms, aiding in the formulation of more tailored treatment strategies. In the realm of gait abnormality rehabilitation training, integrating lower limb exoskeleton technology proves instrumental in helping to ameliorate abnormal gaits, lower the risk of falls, and ultimately elevate the quality of life for patients [5].

Hence, gait-abnormality-recognition techniques can help mitigate the impact of neurodegenerative diseases. The real-time acquisition and analysis of human gait data have undergone extensive exploration utilizing multiple sensor types [6,7,8], primarily categorized as environmental sensors and wearable sensors. Environmental sensors, not requiring direct attachment to the body, are deployed in the surroundings to capture human walking images through screen analysis. The advantage of employing environmental sensors for abnormal gait classification lies in the absence of additional wearable sensors, enhancing user comfort. Moreover, the method of gait classification using an RGB camera is cost-effective, and environmental sensors can capture multi-joint position information, facilitating a comprehensive assessment of user movement. However, environmental sensors have the drawback of requiring users to remain in a fixed position for monitoring, limiting their practicality to hospitals and rehabilitation facilities and rendering them less suitable for daily life assessments. On the other hand, wearable sensors involve placing sensors on various parts of the patient’s body, transmitting data wirelessly to the terminal for analysis [9].

Extensive research has been dedicated to gait monitoring in daily life through wearable sensor technology. For instance, the LCWSnet algorithm proposed by Jing Gao et al. [10] utilizes wearable inertial sensors to capture information on the leg Euler angle. The algorithm uses a Long Short-Term Memory and Convolutional Neural Network (LSTM+CNN) network to model and analyze the Euler angle of legs and realizes the recognition of abnormal gait, Further, by combining with the WhSPP algorithm, automatic feature extraction optimization is realized. Although their proposed method achieved an acceptable 93.1% accuracy in simulations, it is worth noting that their algorithm’s testing solely involves its own simulated data, and only the Euler angle of legs is used to recognize gait abnormalities. Therefore, the universality of their algorithm with respect to real-world applications warrants further scrutiny. Additionally, Kimin Jeong et al. [11] designed a smart insole for classifying in-toe gait, normal gait, or out-of-toe gait. They utilized artificial neural networks (ANNs) trained on sensor data, including accelerometers and gyroscopes, achieving a relatively good recognition rate. However, the applicability of this method to recognizing other gait abnormalities requires additional investigation. Abdullah S. Alharthi et al. [12] employed Layer-wise Relevance Propagation (LRP) in conjunction with a Deep Convolutional Neural Network (DCNN) to classify Parkinson’s severity based on gait-induced ground reaction force (GRF) data. They have a robust recognition rate of 95.5%, but their experiments were only focused on GRF data, which has inevitable limitations in exploring its applicability to other portable sensor data. Ogul C. Yurdakul et al. [13] also addressed the classification of Parkinson’s severity using GRF data by employing the Neighborhood Representation Local (NB-LBP) method for data encoding with a trained ANN network. The study showed a notable enhancement in network recognition rates following data encoding, but there is no further discussion on other widely used recognition networks.

The above-mentioned studies employed portable sensors for a range of gait-abnormality-recognition tasks, which demonstrate the adaptability of these sensors in recognizing abnormalities across diverse environments with impressive accuracy. However, their works cannot guarantee recognition accuracy in environments containing multiple sensor types, such as mobile devices like bracelets, cell phones, or portable pressure insoles, for daily exercise monitoring. Thus, a generalized multi-sensor gait abnormality algorithm is critically important for multiple-type sensors using data fusion techniques. For instance, Aite Zhao et al. [14] integrated neurodevelopmental diseases (NDDs) and assessed disease severity to analyze the multi-modal gait feature by integrating the NDDs and assessing disease severity using a multi-sensor data fusion approach named Correlation Memory Neural Network (CorrMNN). Their CorrMNN could effectively find the correlations among multi-channel data. However, the CorrMNN needs a heavy workload in computation to extract abnormal gait features for neural network training and classification. Yunas et al. [15] used a hybrid bio-inspired evolutionary search algorithm and correlation-based feature-selection method and evaluate their impact on extracted feature vectors from individual sensor modality. The above studies are summarized in Table 1 for comparison.

The aim of this article is to propose a framework that is applicable to a wide range of portable sensors and has good accuracy in different gait-abnormality-recognition classifications. When dealing with data collected by portable sensors, we usually face several problems: (1) high-frequency noise may interfere with recognition learning due to communication noise and shake; (2) users’ step lengths and speeds may result in inconsistent data lengths due to differences in disorder and personal habits, increasing computational costs; (3) smooth data interfered with the fact that smooth data from standing did not show the specificity of gait abnormalities, and the start and end times were difficult to standardize; (4) inconsistency of data types and different types of portable sensors may produce different data types that need to be represented uniformly for identification and classification; (5) the generalization of recognition method and too many training parameters may place demands on training time and equipment performance, limiting the generalization and scope of application of the algorithms.

To tackle these challenges, we introduce a multimodal gait-abnormality-recognition framework based on a CNN-LSTM network, designed to be versatile across various portable sensors and achieve the high-accuracy recognition of multiple abnormal gait patterns. In acquiring multi-sensor data, we utilize a Butterworth filter and an adaptive sliding window for data preprocessing. The adaptive sliding window method selects and filters data based on their characteristics, controlling data length and enhancing the dataset by eliminating smooth data. Subsequently, depending on the time–frequency characteristics and type of data, the preprocessed data are transformed into image sequences using wavelet transform or gray value mapping. This step aims to extract features from the data and provide a standardized representation of different data types for subsequent training, laying the groundwork for network training. Finally, the obtained image sequences are trained using a CNN-BiLSTM recognition network. The network leverages the CNN’s spatial feature extraction capability and the LSTM network’s modeling performance to learn features from the image sequences. In the training process, only the CNN network undergoes pre-training to reduce the number of parameters to be trained. To validate the recognition performance of the framework, we obtained three benchmark datasets from PhysioNet and simulated a gait abnormality dataset using a smart bracelet. The performance of the recognition framework in various recognition tasks was assessed by conducting diverse classification tasks on these four datasets. The experimental results were evaluated using performance metrics, including accuracy, confusion matrix, and F1-score. These metrics demonstrate that the algorithm surpasses the majority of recognition algorithms in tasks such as Parkinson’s disease severity classification and neurological disorder gait abnormality classification. The framework achieves high-accuracy recognition without an excessive number of training parameters and is capable of leveraging data from multiple sensors. This makes our framework suitable for daily health monitoring and has a promising future in rehabilitation areas such as lower-limb exoskeleton and fall risk assessment.

This paper is organized as follows. First, the importance of gait-abnormality-recognition techniques in the context of increasing neurological diseases due to increased aging is presented, and research related to gait abnormality recognition using portable inertial sensors is discussed. In Section 2, we describe the dataset used and detail the recognition framework. In Section 3, different recognition classification tasks are described using the dataset and compared with existing recognition algorithms to verify the superiority of the algorithm proposed in this paper. Section 4 summarizes the paper and presents future research directions and application outlook.

## 2. Methods

This section has five main parts: (1) an overview of the components of the recognition framework; (2) data acquisition; (3) data preprocessing; (4) feature extraction; and (5) the recognition network. This section describes in detail the implementation of the components of the recognition framework and the roles they play.

### 2.1. Framework Structure

**Figure 1:**
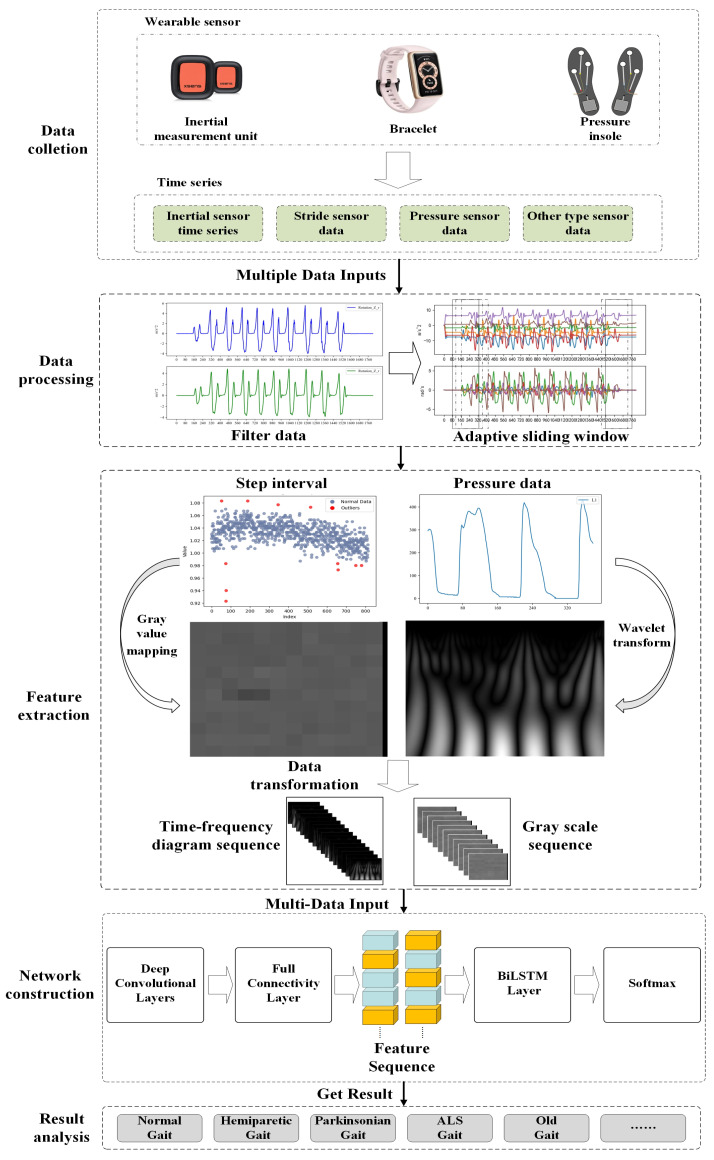
Structure of the multi-sensor data- fusion gait recognition framework. The framework contains three main components: preprocessing, feature extraction, and recognition network. It is applicable to a wide range of abnormal gait-recognition tasks and is capable of handling different types of sensor data.

The proposed multi-sensor pattern fusion for gait abnormality recognition consists of three parts, as shown in Figure 1. (1) Data preprocessing: use the Butterworth filter and adaptive sliding window method to reduce noise and avoid excessively long data series. (2) Feature extraction: use the wavelet transform and gray value mapping method to extract features from the data and convert different types of data into time–frequency image sequences in a unified way. (3) Recognition network: utilize the CNN-BiLSTM network trained to recognize time–frequency image sequences and achieve recognition of abnormal gait caused by multiple neurological diseases.

### 2.2. Data Collection

To validate the framework in different sensor recognition tasks and its effectiveness in gait recognition for neurodegenerative diseases, four datasets were collected for testing and training, three of which are from PhysioNet.

#### 2.2.1. Gait in Parkinson’s Disease Database(PDgait Dataset)

This dataset was used for a dichotomous classification task for Parkinsonian gait. A total of 93 patients with idiopathic Parkinson’s disease (mean age: 66.3 years; 63% men) and 73 healthy controls with gait measurements were collected in the dataset. The acquisition equipment comprised eight sensors (Ultraflex Computer Dyno Graphy, Infotronic Inc., Northville, MI, USA) placed under each foot, through which the recordings of vertical ground-reaction forces were obtained when the tester walked for 2 min at a self-selected speed at the sensor sampling frequency of 100 (sample/s). In addition, the dataset contains demographic information as well as measures of disease severity [16].

#### 2.2.2. Gait in Aging and Disease Database

These same data were used to study the gait profile of aging Parkinson’s disease in a dataset that contained sequences of walking intervals for 15 subjects, including 5 healthy young adults, 5 healthy older adults, and 5 older Parkinson’s patients. Subjects walked continuously on a flat surface around an accessible route, with step intervals acquired using resistive sensors placed in the shoe.

#### 2.2.3. Gait in the Neurodegenerative Disease Database (NDDs Dataset)

This is a dataset for a multi-categorization, which acquires data primarily from two pressure sensors placed in the shoe. The database contains records from patients with Parkinson’s disease (15 cases), Huntington’s disease (HD) (20 cases), and amyotrophic lateral sclerosis (13 cases), as well as records from 16 healthy control subjects. Data types include (1) pressure sensor values; (2) elapsed time; (3) stride interval; (4) swing interval; (5) stance interval; (6) support interval [17,18].

#### 2.2.4. Abnormal Gait Simulation Data

This dataset was generated from walking data using the HUAWEI Band 6 (Huawei, Shenzhen, China). We leveraged the Health Industry SDK provided by the company to access gyroscope and accelerometer data embedded in the band, sampled at a rate of 10 ms. Additionally, two bracelet dials were affixed to the sides of each shoe to capture foot movement during walking. This configuration is primarily employed to simulate the following gaits.

- Normal Gait: The foot moves forward during the swing phase with hip flexion, knee flexion, and then extension, accompanied by ankle dorsiflexion, resulting in a shortening of the lower extremity to get the foot off the ground. This is followed by a gradual extension of the knee before the heel hits the ground, followed by a slight flexion of the knee in preparation for the stance phase. Under normal walking conditions, the time sequence of these motor characteristics remains almost constant [19].
- Parkinsonian gait, a chronic disorder commonly seen in Parkinson’s disease; resting tremor; bradykinesia; and rigidity are the main symptoms of Parkinson’s disease [20]. Early-stage Parkinson’s disease patients may present with slowed gait speed, reduced stride length, decreased amplitude of the swing of the arms, and increased bilateral limb asymmetry and gait variability. Patients with mid-stage Parkinson’s disease may show symptoms in both limbs, with movement becoming more sluggish, along with a corresponding increase in bilateral lower extremity support, and a further decrease in the amplitude of the arm swing. At the same time, there may be abnormal changes in posture, such as leaning forward and the development of a freezing gait and panic gait. Gait disturbances worsen, and motor dysfunction (e.g., freezing gait) becomes more frequent in patients with advanced stages of the disorder, accompanied by decreased balance and postural control and a serious risk of falls.
- Hemiplegic (HP) gait, commonly seen in central nervous system injuries such as stroke, affects mainly one side of the body’s limbs. Early in the swing phase, the hips are elevated and the trunk is swung laterally to raise the foot-to-floor clearance, and trunk and pelvic movements shift the weight to the unaffected side. There is toe dragging or compensatory rounding of the legs, and the sagittal planes are disturbed during the swing phase of the hemiplegic limbs at the hips, knees, and ankles [21].
- Scissor (SC) gait, commonly seen in central nervous system diseases such as cerebral palsy and spinal cord injuries, is a coronal plane pathology [22], because of the spasticity of the hip adductors, which tends to cross the advancing leg over the standing leg during the swing phase [23]. The hips and knees are slightly bent when walking. Both knees rub or bump against each other, sometimes even crossing the legs.

Based on these abnormal gait characteristics, a simulated walk was performed on a straight 5-m route. Considering the differences in step length and the step frequency of actual patients, we constructed the required experimental test dataset by simulating 20 individuals using different step lengths and step frequencies to present the four gait performances. The following section illustrates the implementation of the framework with the processing of this dataset. During the data-collection process, we utilized a Bluetooth protocol to transmit sensor data from the smart bracelet to the smartphone while synchronizing time information, ensuring that the collected data are timestamped. The data are synchronized based on the timestamps and used for subsequent experiments. If an error occurred during data transmission, our monitoring program recorded the time point of the error’s occurrence and the time point of the sensor reconnection, and the data between the two time points were deemed invalid. To avoid the impact of invalid data, the data between the two time points were abandoned.

### 2.3. Data Preprocessing

#### 2.3.1. Filter

When using sensors for the detection of gait abnormality, the dataare inevitably affected by the noise of the sensors themselves and external interference, and the inconsistency of step frequency and stride length of different users leads to different lengths of time for completing the test, so it is necessary to carry out pre-processing to reduce the effect of noise on the recognition accuracy and to convert the data into a dataset that can be used for classification training.

**Figure 2:**
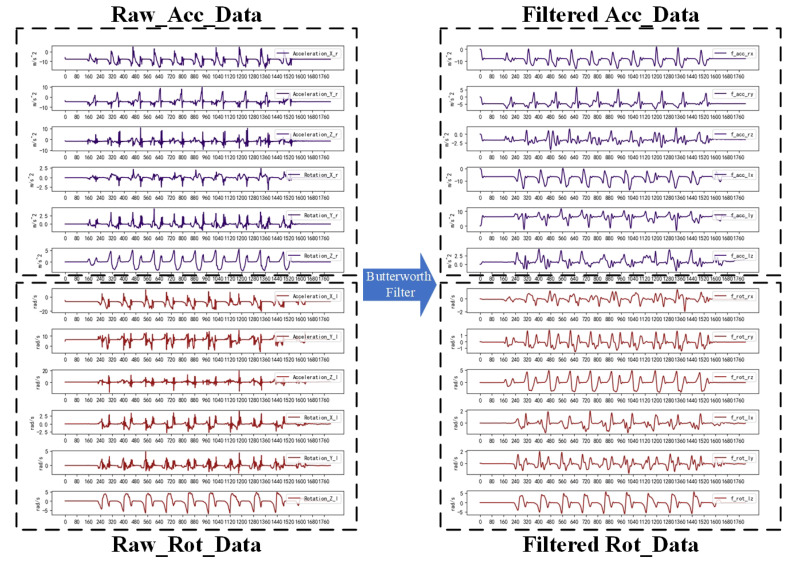
Butterworth low-pass filtering effect: after applying the filter, the high-frequency noise is effectively removed while the phase of the signal is changed.

The data acquisition was performed by HUAWEI Band 6 at 10 Hz; the main data were the 3D accelerometer data and the 3D gyroscope data. The source of noise may consist of jitter during testing, noise from the sensor itself, and noise during transmission. The Butterworth filter is widely used in signal processing, image processing, and other fields due to its flat amplitude–frequency response and the absence of ripple characteristics in the transition region of the stopband. This system selects the Butterworth filter bandpass filter for bandpass filtering, which mainly filters the high-frequency component brought aboutby the noise and the low-frequency component brought about by the jitter. Although the Butterworth filter has a phase delay, it has no effect on the classification detection. The effect of filtering 12 ((3D acceleration + 3D angular velocity) × 2) time series using the Butterworth filter is shown in Figure 2.

#### 2.3.2. Adaptive Sliding Window

Despite the effective noise reduction achieved by the Butterworth filter, the processed data sequence still confronts two challenges: excessive length and an abundance of smooth data. Lengthy time series may come from users with a slow space and will increase the input of the identification network, thus increasing the complexity of the recognition network. Smoothing data usually come from measurements taken at a standstill and unable to demonstrate changes in gait abnormalities. The smoothing data as invalid data will decrease recognition accuracy.

**Figure 3:**
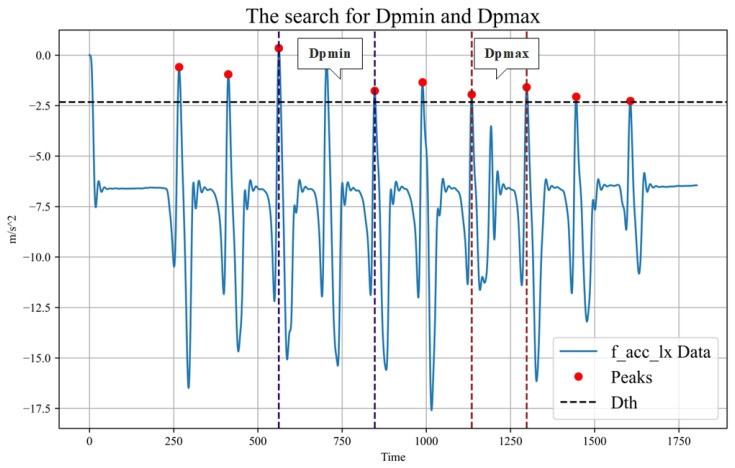
Identifying Dpmin and Dpmax provides an appropriate window length selection for the subsequent sliding window data segmentation.

To address the issue of the smooth data interference and extensive time series in gait abnormality recognition, the framework incorporates the adaptive sliding-window method. The sliding window is a widely adopted data-interception technique offering effective sequence length reduction and data enhancement. The adaptive sliding-window method can adjust the window threshold and window size according to the data characteristics. By using a suitable window size and window thresholds, the method splits long data and filters smooth data. The sliding window size and sliding step size are mainly determined by peak detection and peak distance ordering of the data, with the window thresholds calculated using the standard deviation of the data. The adaptive sliding window method is implemented as follows:

First, the standard deviation (Dstd) is calculated for each type of data. Then, the threshold value (Dth) for that type of data is obtained by multiplying a fixed threshold factor (Df) with the standard deviation. Next, peak detection is performed using the thresholds to obtain the peak value for each data type (Dpeaki). Subsequently, the maximum two consecutive peak distances (Dpmax) and the shortest peak distance (Dpmin) were calculated for each data type. From these, the largest Dpmax is selected as the Gpmax and the smallest Dpmin is selected as the the Gpmin. Gpmax is used as the sliding window size and Gpmin is used as the sliding step size. The finding of Dpmax and Dpmin is shown in Figure 3. The sliding window algorithm is shown in Algorithm 1.
**Algorithm 1** Adaptive Sliding Window 1:**function** AdaptiveSlidingWindow (data,Df,window_threshold) 2:      Dpmax_array=[] 3:      Dpmin_array=[] 4:      subwindow_array=[] 5:      **for** *i* **in** {0,1,…,len(data)−1} **do** 6:            type_data=data[i] 7:            Dsd=cal_std(type_data) 8:            Dth=Df×Dsd 9:            peak_array=find_peaks(type_data,Dth)10:            Dpmax,Dpmin=get_Dp_params(peak_array,2)11:            Dpmax_array.append(Dpmax)12:            Dpmin_array.append(Dpmin)13:      **end for**14:      Gpmax=max(Dpmax_array)15:      Gpmin=min(Dpmin_array)16:      step_size=Gpmin17:      window_size=Gpmax18:      **for** *i* **in** {0,…,len(data)−window_size+1,step_size} **do**19:             subwindow=data[i:i+window_size]20:             **if** process_window(subwindow,window_threshold) **then**21:                subwindow_array.append(subwindow)22:             **end if**23:      **end for**24:      **return** subwindow_array25:**end function**

**Figure 4:**
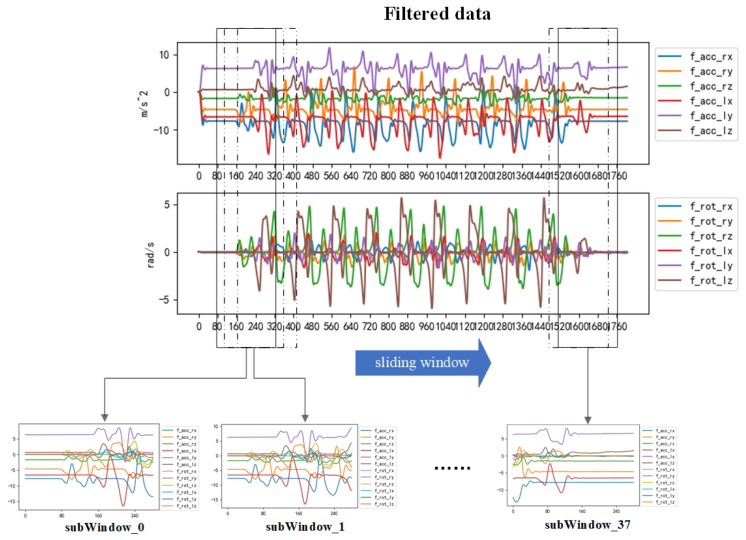
Adaptive sliding- window data extraction. Using this method to segment and filter the data, a total of 38 sub-windows were obtained in the data as shown in the figure.

This method not only preserves the diversity of abnormal gait data in the time–frequency domain but also reduces the data length and enhances the training data, thus effectively improving the training speed and recognition accuracy. Eventually, the raw data collected by the sensor are converted into the dataset intercepted by the sliding window. The effect of data extraction is shown in Figure 4.

### 2.4. Feature Extraction

While obtaining uniformly lengthened dataset post-preprocessing allows for the direct use of these time series in recognition training, it often results in diminished accuracy. Feature extraction from time series can mitigate this by reducing data dimensionality, enhancing classification and regression performance, and improving model interpretability. The approach to feature extraction varies depending on the nature of the time series and the application domain. In handling multi-sensor data, the presence of diverse data types—such as stride intervals, ground reaction forces (GRF), and acceleration sensors—poses a challenge in accurately representing data characteristics through direct concatenation. Hence, the challenge in multi-sensor input problems lies in achieving effective feature extraction while ensuring a unified representation of different data types.

Gait abnormalities often manifest as a slowing of step frequency, inconsistent cycles between legs, and the emergence of compensatory gait. Feature extraction aims to find ways to better capture these scenarios from sensor data, thereby achieving higher recognition rates. In cases of inconsistent leg cycles and compensatory gait, corresponding changes in the frequency and amplitude of sensor data occur. Hence, utilizing the time–frequency characteristics of sensors for feature extraction is a common and effective method. In addition, the discrete Fast Fourier Transform (FFT) combined with Principal Component Analysis (PCA) is used to obtain the features [24], Nyquist frequency [25], and Fourier-transformed spectrogram with shallow features [26]. Wavelet transform is also a typical method for time–frequency feature extraction, and wavelet transform is usually preferred over FFT or short-time Fourier transform (STFT) because of its ability to preserve time-resolved distributional information in the high and low frequencies of the signal, and it therefore has a wide range of applications in time-series prediction and recognition [27,28]. In addition, the extracted gait data are non-smooth, and the time-varying characteristics and transient changes in the gait abnormality signals can be captured using wavelet transform.

In this system, the wavelet transform is used to extract time–frequency features from the original time series such as accelerometers and plantar pressure sensors. According to the data frequency characteristics, different fundamental wave functions are selected for decomposition. For example, when the step frequency is slow, Gaussian wavelet is selected for decomposition in order to obtain good localization, while a Morlet wavelet is chosen when the step rate is fast, to capture a more accurate instantaneous transform. Commonly used wavelet functions are as follows.

- The Gaussian wavelet, which has a smooth shape, can filter certain errors and noise and through parameter adjustment can be made to have better localization in both the time domain and the frequency domain so that it can capture the characteristics of gait abnormality. The fundamental wave function is as follows:
  (1) $$W\left(a,b\right)=\int_{-\infty }^{\infty }x\left(t\right)\cdot \frac{1}{\sqrt{a}}\cdot \frac{1}{\sqrt{2\pi }}\cdot e^{-\frac{{\left(t-b\right)}^{2}}{2a^{2}}}\;dt$$
- The Morlet wavelet, a complex wavelet, consists of the product of a Gaussian function and a complex exponential function. It has the advantage of capturing both amplitude and phase information of the signal and has a high frequency resolution, as the main frequency components in the frequency domain are concentrated near the center frequency. This allows the Morlet wavelets to capture transient changes in the time domain more accurately, and it is suitable for analyzing rapid changes in gait abnormality signals:
  (2) $$W\left(a,b\right)=\int_{-\infty }^{\infty }x\left(t\right)\cdot \frac{1}{\sqrt{a}}\cdot e^{i\omega_{0}\left(t-b\right)/a}\cdot e^{-\frac{{\left(t-b\right)}^{2}}{2a^{2}}}\;dt$$

**Figure 5:**
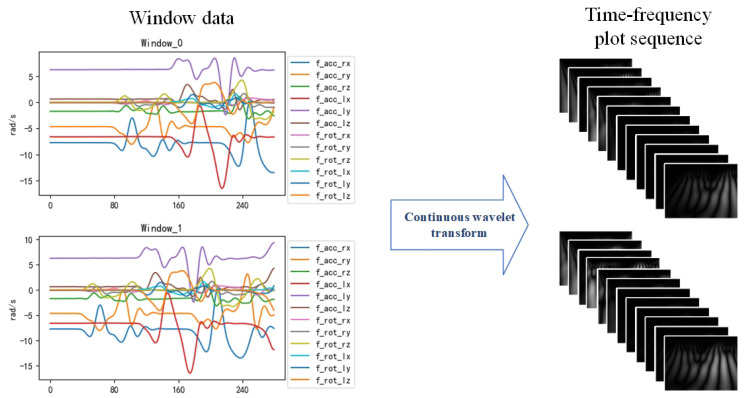
Gaussian wavelet transform feature-extraction effect.

Using wavelet transform, we decompose the original time series into wavelet coefficients, which contain the characteristics of the signal in the time and frequency domains. Then, a time–frequency diagram reflecting the time–frequency characteristics of the data is plotted based on the obtained wavelet coefficients. When gait abnormalities occur, the time–frequency plot will also change accordingly. For example, the wave peaks on the time–frequency plot of the plantar pressure data of Parkinson’s patients will become denser. The effect of choosing a Gaussian wavelet for feature extraction is shown in Figure 5.

**Figure 6:**
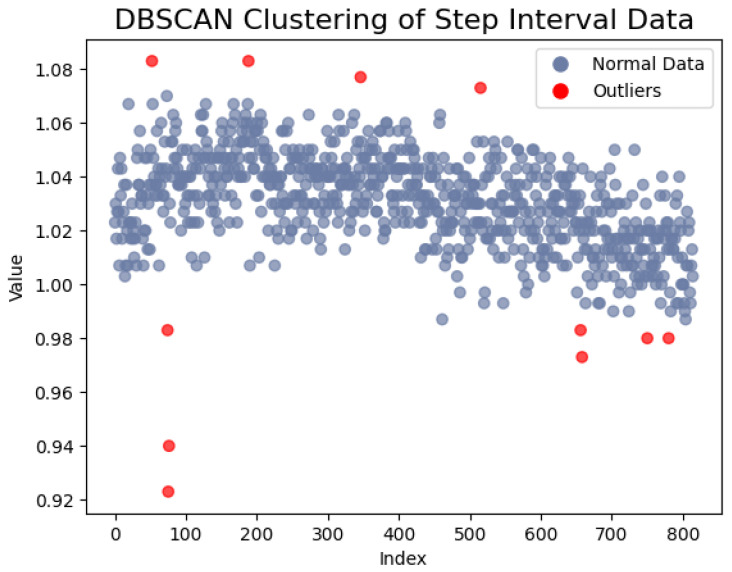
Clustering of the step interval data to reject outliers.

For data with time–frequency characteristics, such as the step interval time and the swing time, we employed gray value-mapping technology to transform the data sequence into images. Prior to the sliding window interception, we utilized Density-Based Spatial Clustering of Applications with Noise (DBSCAN) to eliminate points far from the clustering center and normalized the data sequence. Subsequently, we mapped the normalized data to a grayscale range of 0–255, where the data value corresponds to the intensity of the gray block. Then, a sliding window is involved to intercept the data. This process yields an image encompassing time–frequency characteristics, with each data point represented as a grayscale block, providing a more intuitive depiction of data distribution and variation. When gait abnormalities result in a slowing of the stride, the intensity of the corresponding grayscale blocks decreases. If the gait cycle is inconsistent, the difference between adjacent images in the image sequence will also increase. The DBSCAN clustering schematic is illustrated in Figure 6.

### 2.5. CNN-BiLSTM Recognition Network

The sequence of time–frequency maps is obtained through preprocessing and feature extraction, and these time–frequency maps reflect the time–frequency characteristics of different sensors captured in different dimensions during the walking process. We will analyze the abnormality relationship between the time–frequency maps by building a network to realize the classification and recognition of different abnormalities. Compared to data sequences, time–frequency images provide higher-level, more expressive features that help the machine learning model better capture the time-domain and frequency-domain characteristics of signals. The image data are also easier to enhance, which helps to train more robust models.

**Figure 7:**
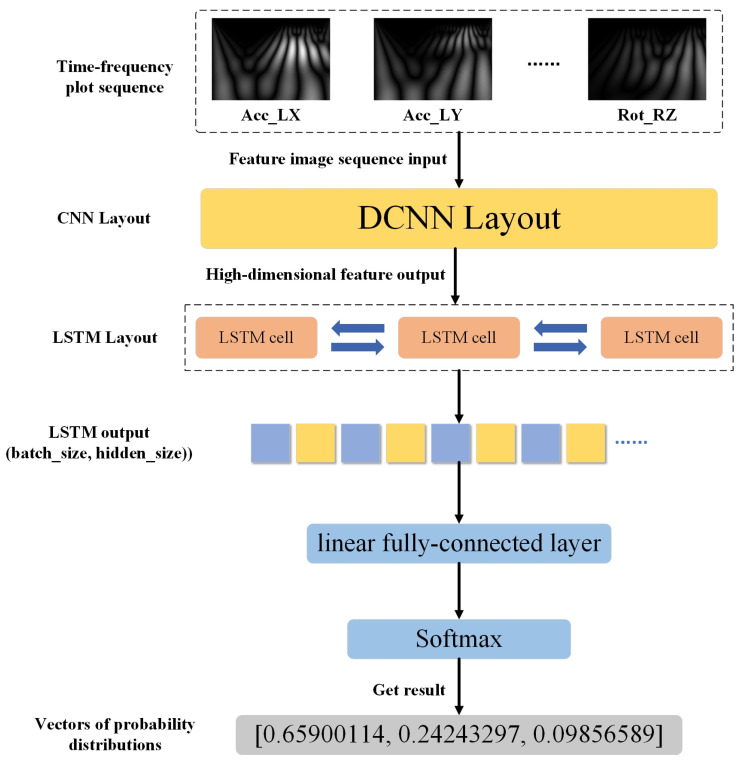
CNN-BiLSTM recognition network framework.

The CNN-LSTM network is a hybrid neural network that has achieved a wide range of applications in several fields, including emotion recognition [29], video action classification [30], pasta product classification [31], facial micro-expression recognition [32], gait recognition [33], and stock prediction [34]. Its main framework is made of a convolutional layer spliced with a BiLSTM layer, and then the attention mechanism and other networks can be added according to the data characteristics and task requirements. Its working mechanism is mainly to extract spatial features from the data using CNN, then networking the extracted feature information using BiLSTM for time series modeling, and finally adding a fully connected layer for classification and recognition tasks. The network architecture is shown in Figure 7.

During walking, gait abnormalities are expressed in the sensing data, and there is a correlation between the data from each sensor, with different abnormal gaits presenting different correlation characteristics. The method proposed in this paper is used to model these correlated features to achieve the recognition of different abnormal gaits. After feature extraction, the sensor data sequence is converted into an image sequence representing time–frequency features. Then, the CNN network extracts the features of these images to obtain the eigenvector. Then, the eigenvectors extracted from different sensors are combined into feature sequences and then input into LSTM network for correlation learning.

#### 2.5.1. Deep Convolutional Network

DCNN is a kind of deep neural network that is widely used in tasks such as target detection, image classification, natural language processing, and so on. It is mainly composed of a convolutional layer, a pooling layer, and a fully connected layer, the core of which is the convolutional layer, which goes through the convolutional kernel of the input image sliding convolution. Therefore, as to extract the local features in the image, the dimensionality of the features after convolution will be very high, and it is necessary to enter the obtained features into the pooling layer for downscaling to extract the main features. DCNN can be very good at capturing the local features of the image through the parameter-sharing mechanism and the pooling operation for localized features of the image. Based on the convolutional feature-extraction logic of DCNN, many improved models have been derived, such as ResNet, VGG, Xception, and DenseNet. Compared with the traditional DCNN network, ResNet solves the gradient problem of the DCNN network in the deep network training by introducing the residual block, which makes the network learn the residual mapping instead of the original mapping during the training process. The problem of gradient vanishing and gradient explosion exists in the DCNN network.

This system is a set of time–frequency map sequences after feature extraction because the output of the time–frequency map is single-channel, so the size of a single time–frequency map is 1 × 340 × 240, and the time–frequency map sequence is input into the same DCNN network in a fixed order, and the spatial feature information contained in the time–frequency maps is extracted to form a set of feature information sequences.

#### 2.5.2. Bidirectional Long Short-Term Memory

LSTM is a special kind of recurrent network. LSTM effectively solves the problem of gradient vanishing and gradient explosion that has existed in RNN for a long period of time in the middle by introducing a gating mechanism [35]. It can effectively solve the problem that the data types may increase exponentially when multiple sensors are used to collect data from different parts of the body. The LSTM unit is mainly composed of four parts: unit state, forgetting gate, input gate, and output gate [36].

By forgetting and memorizing the information in the unit state, the information that is useful for the subsequent moments in the propagation process is transmitted, and the useless information is discarded, and the hidden layer state, ht, is output at each time step. When there is an input to the LSTM unit, the state of the forgetting gate, the input gate, and the output gate is calculated using the hidden layer state ht-1 of the previous moment and the current input.

**Figure 8:**
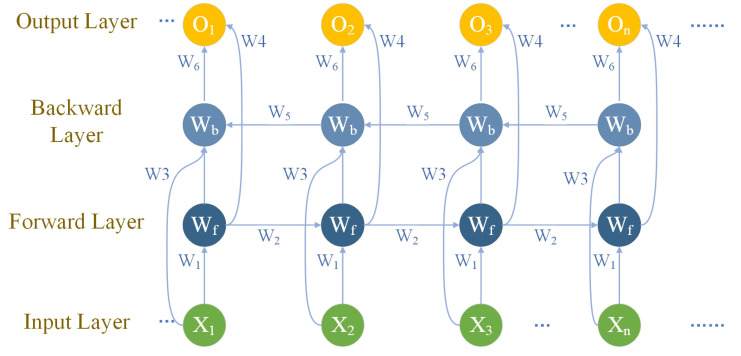
BiLSTM structure.

Bidirectional Long Short-Term Memory (BiLSTM) is an improved type of LSTM: BiLSTM adds a reverse loop to LSTM, which makes it possible to consider both past and future information during the computation process, mitigates information loss, and better captures the contextual relationships of the sequence. Thus, using the BiLSTM will better model the relationships among the feature information. The BiLSTM’s structural network is shown in Figure 8.

#### 2.5.3. Model Evaluation Indicators

In the training phase, the dataset undergoes division into training and testing sets. Following the training of the network’s parameters on the training set, it is applied to predict and classify data within the testing set. Gait abnormality recognition is treated as a multi-classification problem, and for the assessment of the model in such scenarios, metrics like accuracy, normalized confusion matrix, F1-score, Recall, and Precision are widely employed [33]. The specifics of the normalized confusion matrix are outlined in Table 2.

(3) $$\mathrm{Accuracy}=\frac{TP+TN}{TP+TN+FP+FN}$$

(4) $$\mathrm{Precision}=\frac{TP}{TP+FP}$$

(5) $$\mathrm{Recall}=\frac{TP}{TP+FN}$$

(6) $$F1\text{-}\mathrm{score}=\frac{2\times \mathrm{Precision}\times \mathrm{Recall}}{\mathrm{Precision}+\mathrm{Recall}}$$

- TP: number of samples that were actually in the positive category and were correctly predicted to be in the positive category.
- FP: number of samples that were actually in the negative category but were incorrectly predicted to be in the positive category.
- FN: number of samples that were actually positive but incorrectly predicted to be negative.
- TN: Number of samples that were actually negative and correctly predicted to be negative.

## 3. Experiments and Results

In order to evaluate the recognition performance of the framework when using different portable sensors, we conducted a series of tests using four different datasets. Three of these datasets are from the public dataset, using pressure sensors to measure the user as they walk, and the other dataset is from inertial sensor data modeling gait abnormalities. For each of these datasets, we used an adaptive sliding window approach to cut the data, using 80% of the data for training and the remaining 20% for validation. We used PyTorch 2.0.1 and Python 3.9 for our training environment, and some of the parameter settings for training the network in our experiments are shown in Table 3.

### 3.1. Result of Abnormal Gait Simulation Data

**Figure 9:**
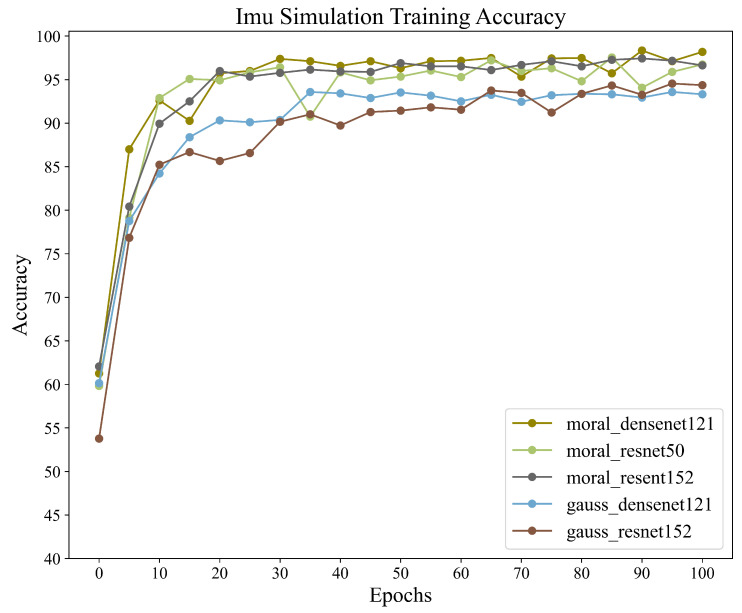
Training accuracy of the IMU dataset with different CNN-BiLSTM structures.

For the classification of gait abnormality in neurological diseases, this dataset, which models neurologically abnormal gait, we aimed to train and recognize this dataset using different combinations within the CNN+BiLSTM framework in order to obtain the accuracy of different combinations in this framework. We also wanted to validate the potential of the framework for application when dealing with inertial sensor data by training it on this dataset. A total of five different sets of wavelet transform and CNN networks were used to train the same dataset for this experiment. All data were obtained via adaptive sliding window interception. The training results are shown in Table 4, and the accuracy graph during training is shown in Figure 9.

**Figure 10:**
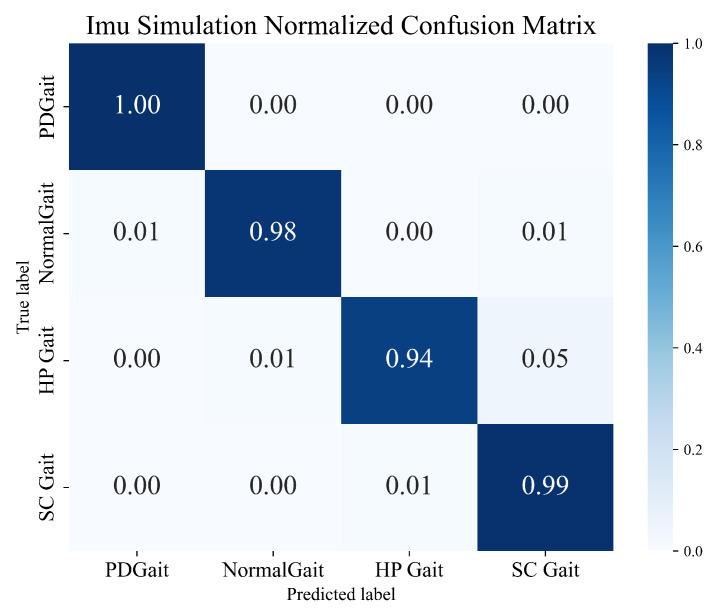
Normalized confusion matrix for the gait-abnormality-recognition task with the IMU dataset.

The training results show that different training combinations of the framework exhibit excellent recognition performance in multi-class gait-abnormality-classification tasks. In particular, for the Moral+Dense121+BiLSTM combination, which achieves a recognition accuracy of 98.35%, we notice that the limit of the upper bound of the recognition accuracy is mainly limited by the chosen form of the wavelet transform, while the choice of CNN has less of an impact on it. This is consistent with the fact that CNNs are mainly used for high-dimensional feature extraction and are not involved in classification training. The above results validate that the framework shows good performance and potential in gait-abnormality-classification tasks using inertial sensors. Figure 10 illustrates the normalized confusion matrix obtained from the combination of Moral+Dense121+BiLSTM.

### 3.2. Results on the Gait in the Parkinson’s Disease Database

**Figure 11:**
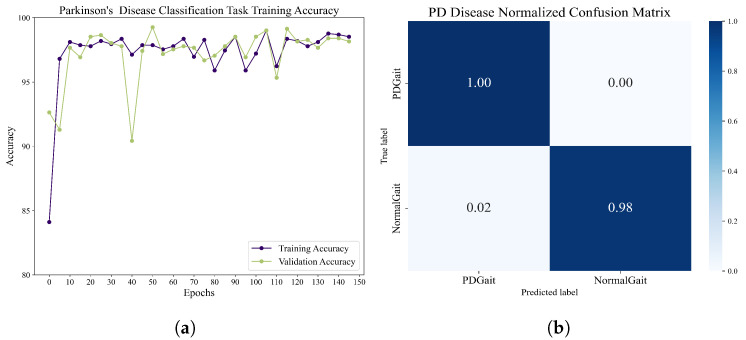
(**a**) The accuracy of the network over time on the test and training sets when performing the Parkinson’s-disease-recognition task. (**b**) The normalized confusion matrix of the network during the Parkinson’s disease-detection task.

The classification of Parkinson’s patients and healthy people revealed the following results. The dataset contains 93 Parkinson’s patients and 73 healthy controls. The dataset was recorded using 16 plantar pressure sensors at a rate of 100 samples per second, and 24,395 healthy user data and 52,291 Parkinson’s patient data were obtained using the adaptive sliding-window method, for which we use a fourth-order morl wavelet transform to extract features, and a combined structure of densenet121 and BiLSTM was used for training. The hidden layer of BiLSTM contains 256 units, the batch size is set to 32, and the learning rate is 0.01. After 150 epochs of training, the network achieves a 99.0172% recognition rate on the task of classifying Parkinson’s patients from healthy controls. The accuracy and normalized confusion matrix during training are shown in Figure 11. Through training and validation, the framework shows very promising performance in distinguishing between Parkinson’s patients and healthy users.

**Figure 12:**
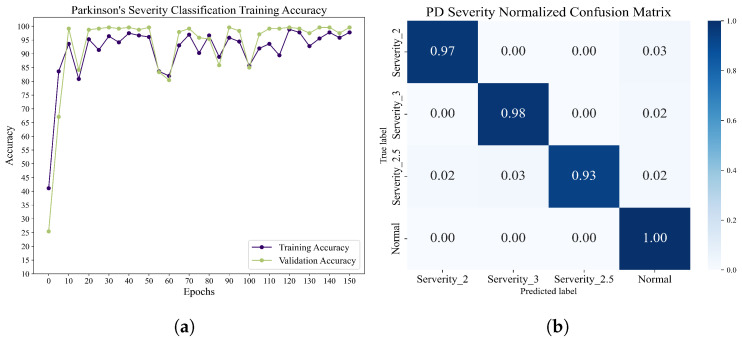
(**a**) The change in accuracy of the network when performing a Parkinson’s disease severity classification task. (**b**) The normalized confusion matrix of the network when performing the Parkinson’s disease severity classification task.

Classification of different Parkinson’s disease severity levels: this dataset is based on the Hoehn and Yahr grading system, which annotates the severity levels of Parkinson’s disease testers into severity level 2, severity level 2.5, and severity level 3. We used the structure of the training network previously applied to the Parkinson’s vs. health classification task. After 100 epochs of training, the accuracy and normalized confusion matrices were obtained, and these results are displayed in Figure 12.

Table 5 demonstrates the accuracy of various algorithms in performing Parkinson’s disease severity classification. The accuracies of other reported BiLSTMs and DCLSTMs are 91.58% and 96.71%, respectively, while LSTM-CNN can achieve a higher accuracy of 97.48%. Remarkably, our proposed recognition framework for Parkinson’s disease achieves the highest accuracy at 98.89%. In Table 6, the accuracy rate of each component under different epochs and the overall F1-score, Recall, and Precision are recorded. It can be found that when the CNN network is only pre-trained, the accuracy rate and F1-score of the method proposed in this paper are low, and it will be heavily biased toward the CO category. However, after 10 epochs training, the accuracy and other evaluation parameters are greatly improved, which proves that the method can reach a higher recognition level with fewer training times. In addition, after 20 epoch training times, Recall reached 98.33%, indicating that the method performed well in recognizing the severity of each category, and the possibility of misclassification into other categories was low.

### 3.3. Results on the Gait in Aging and Disease Database

For the categorization of elderly Parkinson’s patients with different age groups, this dataset was obtained from an ultrathin force-sensitive resistor placed inside a shoe to acquire data from subjects while walking. The difference is that the pressure data from the force-sensitive resistor were converted into a walking step interval time series in the dataset. This section aims to validate the classification performance of the framework for different types of time series by identifying and classifying the stride interval time series.

**Figure 13:**
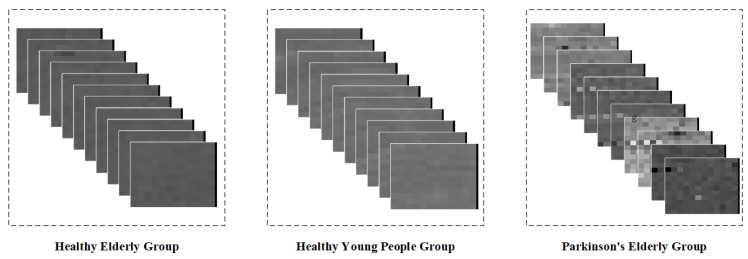
Plot of the effect of mapping the step interval data to grayscale values.

The dataset contains measurement data from five healthy young subjects, five healthy elderly subjects, and five elderly Parkinsonian subjects. Since the data were transformed into step interval time series, they can only be intercepted using a fixed-length sliding window. In addition, wavelet transforms of step intervals do not extract spatio-temporal properties well. Therefore, in this study, we chose the method of gray value coding to graphically represent the step interval time series. Specifically, we mapped the step interval time into grayscale pixels based on the interval duration, thus converting the data from the sliding window into grayscale pixel pictures. Given that this dataset contains only one step-interval element, we randomly sampled the obtained gray-scale pixel pictures of the same type in order to form a sequence containing 10 pictures per group, and the gray-scale map sequence is shown in Figure 13.

**Figure 14:**
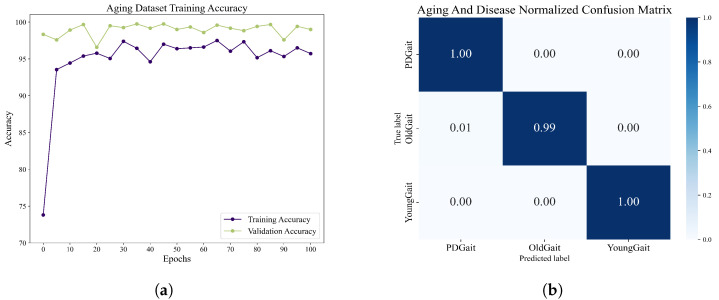
(**a**) The accuracy variation of the method proposed in this paper when recognizing Parkinson’s disease using gait intervals as the input. (**b**) The normalized confusion matrix of the network when recognizing Parkinson’s disease using gait intervals as input.

We obtained 1000 sets of grayscale image sequences of each type through random sampling and used the network of DenseCNN-BiLSTM. After 100 epochs of training, we obtained the accuracy rate as well as the normalized confusion matrix, as shown in Figure 14. The overall maximum accuracy is 97.50%. The experiments demonstrate that the network shows good classification ability when dealing with step-interval sequences, thus demonstrating the potential of the network to be applied to a wider range of situations.

### 3.4. Results on the Gait in the Neurodegenerative Disease Database

Due to potential equipment issues during data acquisition, certain data from a group of Huntington’s disease subjects were excluded as they exhibited abnormality. Our training and testing datasets consisted of data from 16 healthy control subjects, 13 amyotrophic lateral sclerosis patients, 19 Huntington’s disease patients, and 15 Parkinson’s disease patients. To facilitate training, we employed a data-mapping approach reminiscent of the one utilized in the Aging and Disease dataset. This technique enabled the conversion of seven data types, which encompassed left step intervals, right step intervals, left pendulum intervals, right pendulum intervals, left stance intervals, right stance intervals, and correct posture intervals, into image sequences. In total, we generated 410 sets of image sequences.

**Figure 15:**
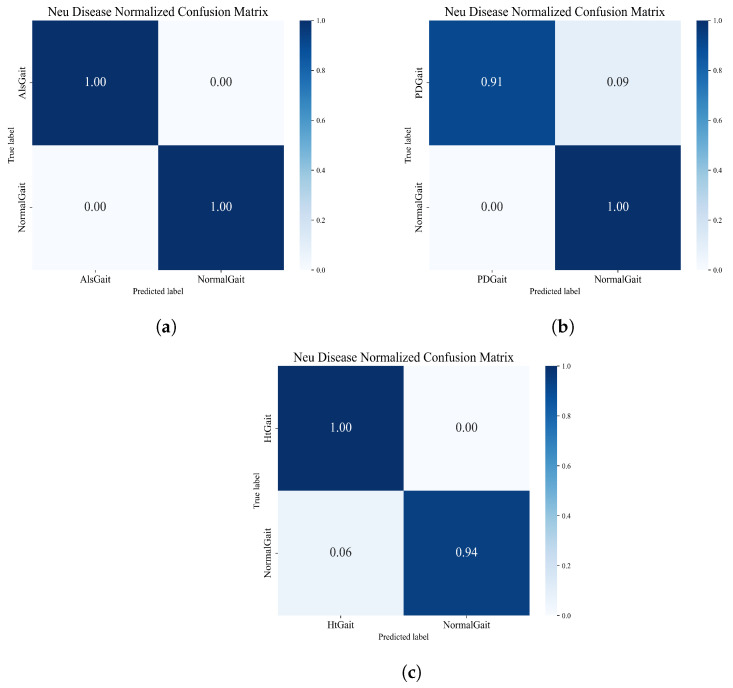
(**a**) The normalized confusion matrix for the ALS-detection task. (**b**) The normalized confusion matrix for the PD-detection task. (**c**) The normalized confusion matrix for the HD-detection task.

For neurological disease recognition, the performance of the framework for neurological disorder recognition was tested by comparing different types of neurological disorder sequences with healthy sequences individually. In this experiment, we set up three different groups. The normalized matrices obtained from the three groups of experiments are shown in Figure 15, which shows that the framework also shows good performance in the task of neurological disease recognition.

We compared the accuracy obtained in the experiment with other advanced methods, and the comparison results are shown in Table 7. As can be seen from the table, compared with traditional machine learning methods such as SVM and RBF, deep learning performs better in recognition accuracy, especially in HD detection. The accuracies of deep learning algorithms are all above 90%, while the accuracies of traditional machine learning methods only achieve a highest accuracy of 88.67%. The algorithm proposed in this paper achieved good accuracy in the detection of ALS and HD, achieving 100% and 96.97% accuracy, respectively, to identify ALS and HD diseases better than other deep networks. Although the PD recognition of this model is lower than that of the DCLSTM model, the accuracy of 95.56% is close to that of other deep learning networks. In addition, it can be seen from Table 8 that the method proposed in this paper achieves satisfactory F1-score, Recall, and Precision when performing the task of classifying neurological diseases, which indicates that the method proposed in this paper has excellent performance in effectively identifying and classifying neurological diseases and possesses high comprehensive performance.

In the above experiments, we applied the framework on the PDgait dataset, NDD, the Aging and Disease database, and simulated gait abnormality dataset and compared it with other state-of-the-art models. Our experimental results show that the framework achieves good accuracy in a variety of gait-abnormality-recognition tasks. For example, in the Parkinson’s-severity-classification task on PDgait, it achieved 98.89% accuracy better than 96.71% for the DCLSTM model and 97.48% for the LSTM+CNN model. In addition, ALS and HD detection on NDD also achieved 100% and 96.97%, respectively. Moreover, compared to the CorrMNN algorithm or the integrated algorithm, the method only trains the BiLSTM, while the CNN performs pre-training. This reduces the demand for computational resources and makes it more suitable for implementation on embedded systems, facilitating the implementation of quantitative gait analysis in daily life.

Although the framework achieves excellent abnormal-gait-recognition results on three benchmark datasets, it demonstrates its application potential in this field. However, because patient data are not public, there is a large gap in the number of datasets used and the clinical data. Therefore, in the follow-up work, we urgently need to expand the abnormal gait dataset and train the framework more adequately to further validate its adaptability and reliability in real clinical scenarios.

## 4. Conclusions

In summary, we constructed a multimodal gait-abnormality-recognition framework based on the CNN-BiLSTM network to accomplish the reduction in long data and the filtering of smooth data using the adaptive sliding window method. In the framework, the time–frequency characteristics of time series of different data types are extracted by applying wavelet transform and gray value mapping, which have a more uniform in representation of different types of sequences. The abnormal characteristics of gait are further extracted using a pre-trained Deep Convolutional Neural Network for feature extraction. Through the processes of multi-sensors input data fusing and classifying, the as-established CNN-BiLSTM network can achieve high-accuracy recognition and is able to be applied for different types of sensors, which reduces the training parameters and achieves high-accuracy gait abnormality recognition. This provides greater flexibility in the practical applications of the gait abnormality reorganization technology without the limitation of specific sensor types and testing environments. By conducting gait-abnormality tasks, including Parkinson’s disease severity classification and degenerative neurological disorder identification, we compare our framework with other reported recognition algorithms to demonstrate the outstanding performances of our proposed framework in various gait abnormality tasks. Overall, the proposed method offers a valuable contribution to the treatment of neurological diseases, addressing the limitations of existing approaches and providing a more effective solution for quantitative gait analysis.

In future research, gait-abnormality-recognition techniques shall be integrated with the Internet of Things (IoT) for the comprehensive monitoring of daily life, and we will combine our proposed method with lower limb exoskeleton control, offering thorough, customized treatment and rehabilitation support for patients with gait abnormalities, thereby mitigating the negative impacts of the disorder on their lives.

## Figures and Tables

**Table 1:** Research on gait abnormality recognition algorithms based on portable sensors.

Recognition Algorithm	Applications	Advantage	Limitations
LCWSnet [10]	Classification of gait patterns such as hemiplegic, tiptoe, and cross-threshold	Combined with the WhSPP algorithm to achieve automatic feature extraction & good recognition rate in abnormal gait recognition.	Multi-sensor input situation cannot be handled, Universality in doubt
ANN [11]	Classification of gait patterns such as internal toe gait, normal gait, or external toe gait	Designed a sensor insole for non-invasive gait anomaly recognition	There is a lack of discussion on the recognition of other abnormal gaits, and it is easy to have cumulative errors
NB-LBP+ANN [13]	Parkinson’s disease detection and severity rating	Effective Parkinson’s severity classification recognition with fewer training parameters	More sensitive to window size selection, limited ability to capture features for multiple data
DCNN+LRP [12]	Parkinson’s disease detection and severity rating	Effective use of feature coding improves recognition accuracy	When the target training data are not balanced, they cannot be recognized well
CorrMNN [14]	NDDs identified, Parkinson’s Disease detection and severity rating, user identification	The recognition of various gait abnormality tasks is realized effectively	Too many training parameters to limit application for daily life monitoring
Ensemble algorithms [15]	Human activity detection	Effective use of correlation for feature selection for different types of task recognition	Too many training parameters to limit application for daily life monitoring

**Table 2:** Normalized confusion matrix definition.

	Predicted Positive	Predicted Negative
**Actual Positive**	$\frac{TP}{FP\;+\;FN}$	$\frac{FN}{TP\;+\;FN}$
**Actual Negative**	$\frac{FP}{FP\;+\;TN}$	$\frac{TN}{FP\;+\;TN}$

**Table 3:** Partial parameters of training network.

Parameters	Value
Lstm hiddern size	256
Initial learning rate	0.01
Batch size	16
Dim of the Conv FC output	512
Optimizer	Adam
Criterion	CrossEntropyLoss

**Table 4:** This table shows the evaluation index of different methods in the Parkinson’s disease-severity-classification task.

CWT	CNN	LSTM	Accuracy	F1-Score
Moral	Dense121	BiLSTM	98.35%	94.53
Moral	ResNet50	BiLSTM	97.54%	93.56
Moral	ResNet152	BiLSTM	97.43%	93.39
Gauss	Dense121	BiLSTM	93.58%	91.73
Gauss	ResNet152	BiLSTM	94.54%	92.18

**Table 5:** This table shows the accuracy of different methods in the Parkinson’s-disease-severity-classification task.

Methods	Accuracy	Methods	Accuracy
CapsNet [37]	95.01%	BiLSTM	91.58%
HMM	93.20%	Q-BTDNN [38]	93.10%
CNN	82.86%	DCCA	94.30%
LSTM+CNN [39]	97.48%	Original LSTM	91.13%
GRU	92.53%	DCLSTM [40]	96.71%
KCAA	92.48%	**CNN-BiLSTM**	**98.89%**

**Table 6:** The following table shows the performance indicators of the CNN+BiLSTM network over different time periods in the Parkinson’s-disease-severity-classification task.

Severity Level	Epoch 0	Epoch 10	Epoch 20
Co accuracy	100%	100%	100%
Severity 2 accuracy	100%	100%	100%
Severity 2.5 accuracy	0%	93.33%	96.66%
Severity 3 accuracy	0%	44.26%	98.36%
All accuracy	25.417%	84.167%	98.75%
F1-score	9.40%	94.12%	98.31%
Recall	24.16%	94.16%	98.33%
Precision	5.84%	94.78%	98.38%

**Table 7:** This table shows the accuracy of different methods in the neurological-disease-classification task.

	Methods	ALS vs. CO	PD vs. CO	HD vs. CO
**Traditional ML**	Multi-SVM [41]	56.66%	51.00%	54.28%
	RBF+DL [42]	89.66%	87.10%	83.33%
	QBC [43]	100%	80%	71.43%
	Meta classifiers [44]	96.13%	90.36%	88.67%
**Deep Learning**	DCLSTM [40]	97.43%	97.33%	96.71%
	PCA+CNN [45]	100%	94.21%	94.98%
	ConvLSTM+QR [46]	97.68%	94.69%	95.05%
	NDDNet [47]	100%	94%	97%
	**CNN-BiLSTM**	**100%**	**95.56%**	**96.97%**

**Table 8:** This table shows other evaluation measures of the method proposed in this paper for the neurological disease classification task.

Model Evaluation Index	ALS vs. CO	PD vs. CO	HD vs. CO
F1-score	100%	95.71%	98.31%
Recall	100%	95.58%	96.67%
Precision	100%	96.15%	96.82%

## Data Availability

The three public datasets used in this paper are available by visiting PhysioNet at https://physionet.org/ (accessed on 20 September 2023). And the datasets simulated by Imu need to be obtained by contacting the corresponding author at the following dataset link: https://github.com/GD-Lws/Imu_sim_data (accessed on 20 September 2023).
